# Vaccine financing in Nigeria: are we making progress towards self-financing/sustenance?

**DOI:** 10.11604/pamj.supp.2017.27.3.11528

**Published:** 2017-06-21

**Authors:** Olumide Faniyan, Chidiabere Opara, Akinyede Oyinade, Pamela Botchway, Kenneth Soyemi

**Affiliations:** 1 Department of Pediatrics, John H Stroger Hospital of Cook County, 1901 W Harrison Street, Chicago, IL 60612, United States

**Keywords:** Novel, financing, vaccine, Nigeria

## Abstract

Nigeria has an estimated population of 186 million with 23% of eligible children aged 12-23 months fully immunized. Government spending on routine immunization per surviving infant has declined since 2006 meaning the immunization budget needs to improve. By 2020, Nigeria will be ineligible for additional Global Alliance for Vaccination and Immunization (Gavi) grants and will be facing an annual vaccine bill of around US$426.3m. There are several potential revenue sources that could be utilized to fill the potential funding gap, these are however subject to timely legislation and appropriation of funds by the legislative body. Innovative funding sources that should be considered include tiered levies on tele-communications, airline, hotel, alcohol, tobacco, sugar beverage taxes, lottery sales, crowd-sourcing, optimized federal state co-financing etc. To demonstrate monthly income that will be derived from a single tax revenue source, we modelled using Monte Carlo simulation trials the Communication Service Tax that is being introduced by the National Assembly. We used number of active telephone subscribers, penetration ratio, monthly charges, and percent of immunization levy as model scenario inputs and dollars generated monthly as output. The simulation generated a modest mean (SD) monthly amount of $3,649,289.38 ($1,789,651); 88% certainty range $1,282,719.90 to $7,450,906.26. The entire range for the simulation was $528,903.26 to $7,966,287.26 with a standard error of mean of $17,896.52. Sensitivity analysis revealed that percentage of immunization levy contributed 97.9 percent of the variance in the model, number of active subscribers and charges per month contributed 1.5%, and 0.6% respectively. Our modest simulation analysis demonstrated the potential to raise revenue from one possible tax source; when combined, the revenue sources will potentially surpass Nigeria’s long-term financing needs. The ROI of vaccine should supersede all other considerations and prompt urgent activities to cover the impending finance coverage gap.

## Commentary

Nigeria’s population is estimated at 186 million with approximately 23% of eligible children aged 12-23 months fully immunized. These sub-optimal vaccine uptakes correlates with elevated risk of vaccine-preventable diseases incidence and outbreaks. National Program on Immunization (NPI) is administered in the National Primary Health Care Development Agency (NPHCDA), a parastatal institution under the Federal Ministry of Health. NPI is financed by contributions from Federal and State governments; in 2015, the total expenditure on immunizations in Nigeria was US$302,100,133 with the federal government contributing US$120,829,723 (40% of the total) leaving a potent funding coverage gap of 60%. There are four sources of finance for immunization services in any given country: 1) Domestic public-funds derived from taxation or other sources of public revenues at the central and/or subnational level, and allocated through a formal budgetary process; this may be current spending, or domestically or internationally held loans, which imply future spending; 2) Domestic private-resources from households, employers, and/or local philanthropists; 3) External public-official development assistance, typically funds derived from taxation in donor countries, allocated per the policies and practices of bilateral and multilateral international aid agencies; this includes the grant (also called concessionary) portion of development loans offered at below-commercial interest rates, and 4) External private resources from international philanthropists [1]. In Nigeria, there are multiple non-governmental funding sources which include personal and private sector donations, and other financing forms such as the Global Alliance for Vaccination and Immunization (Gavi). Financial support for delivering new vaccines to Nigeria is important because it serves a dual purpose of paying for vaccine and encouraging vaccine research, development, and production. By pooling the demand from developing countries for new vaccines and providing long-term, predictable financing to meet this demand, the Vaccine Alliance's business model influences the market for vaccine. The Gavi model has brought “added value” by providing solutions to vaccine availability barriers and laying the foundations that will allow governments to continue immunization programs long after Gavi support ends in 2020 [1, 2]. Increasing infant population, the higher cost of the new vaccines and the declining support from Gavi due to graduation, will increase funding requirement for vaccine program in Nigeria to 450 million dollars by 2020 [3]. Nigeria has initiated several funding mechanisms such as the Public Health Fund which was established by the National Health Law in February 2014 (to be financed by contributions from federal and state revenues) and the Nigerian National Immunization Financing Task Force (NIFT) established in 2015. One of the NIFT’s main directives is to establish an external public-private partnership Immunization Financing Trust Fund (IFTF) to complement the Public Health Fund. Nigeria is not in the top ten African countries with full Dtap 3 coverage, this explicates the importance of innovative financing models to fund vaccine availability and cover the financing vacuum. The return on investment (ROI) on immunization during a 10-year period (from 2011 to 2020) has been calculated to yield a 16-times greater return in averted illness costs, money that can be spent on other competing priorities [4].

From 2006 through 2014, Nigeria’s Gross National Income rose from US$840 to $2,970 per capita, a 254 percent increase; the Nigerian government spent $17 on routine immunization per surviving infant in 2006 compared with $8 per infant in 2014. In addition, the government share of total routine immunization expenditures dropped from 87 percent to 24 percent over the same period. Since 2010, Nigeria has reported inconsistently on JRF financial indicators. The data suggest that Nigeria has not progressed towards country ownership of its immunization program [5] leaving potentially millions of children not fully vaccinated. Nigeria has the largest economy in Africa and the 26th largest in the world, with a GDP of $510 billion. Only 14% of GDP is from the Oil sector, with retail and wholesale trade being the biggest drivers of GDP growth. Nigeria has the potential to achieve 7.1% annual GDP growth which would make Nigeria a top-20 economy in 2030, with GDP of more than $1.6 trillion [6]. Despite Nigeria’s economic growth and potential, one of the key concerns of GAVI model has been sustainability around the issues of local sustenance of vaccine supply and access when GAVI support ends [7]. Nigeria’s government immunization budget needs to increase from its current $145m to $315m in 2020. For vaccines alone in 2020, the government must raise $265m. By the year 2020, Nigeria will be ineligible for any more Gavi grants and will be facing an annual vaccine bill of around US$426.3m. The 2016 National Health Act was an important milestone; however, it is not expected to be fully operational until 2018 and will not provide all the funding needed. For this reason the NIFT is focusing on creating and financing a new national trust fund for immunization [8]. Co-financing by the Federal, State and local governments has been proposed as a viable option with the Federal government paying 52% of the vaccine bill and states progressively paying into the trust fund until reaching their full population- and income-weighted shares in 2021 [8]. There are several potential revenue sources that could be utilized to fill the potential funding gap, these are however subject to timely legislation and appropriation of funds by the legislative body. Innovative funding sources that should be considered to supplement federal and local funding include tiered levies on telecommunications, airline, hotel, alcohol, tobacco, sugar beverage taxes, lottery sales levy, crowd-sourcing, optimized federal state co-financing etc. These revenue sources after studies (feasibility, optimization, and budget impact) will reveal potential sources of maximum revenue to sustain Nigeria’s long-term vaccination financing and operations goals. To demonstrate potential monthly income from one of the aforementioned tax revenue source, we simulated (Monte Carlo simulation trials) using the new Communication Service Tax (CST) that is being introduced by the National Assembly in Nigeria. The CST will be levied on service fees payable by users of electronic communication services at 9% and will be borne by the customers. If the Bill is enacted into law, it will mandate service providers to file monthly tax returns with the Federal Inland Revenue Service with strict penalties for non-compliance [9]. There are over 150 million cell phone users (with 83 per 100 subscribers) and 86 million internet users with average monthly phone bill of $6.00 [10]. For the simulation, we used number of active subscribers, penetration ratio, monthly charges, and percent of immunization levy as model as scenario inputs. The output generated was the amount of dollars generated monthly with certainties. The simulation generated a modest mean (SD) monthly amount of $3,649,289.38 ($1,789,651); 88% certainty range $1,282,719.90 to $7,450,906.26. The entire range for the simulation was $ 528,903.26 to $7,966,287.26 with a standard error of mean of $17,896.52. The sensitivity analysis of the simulation model revealed that percentage of immunization levy contributed 97.9 percent of the variance in the model, while number of active subscribers and charges per month contributed 1.5% and 0.6% respectively. Penetrance ratio did not appear to contribute to the variance. It should be noted that these estimates are modest estimates and may be under or overestimated. It is significant to note that the distinction between public and private sources is somewhat artificial; tax revenues, while collected and administered by public agencies, are fundamentally the product of individual citizens’ labor and ownership of property. While the Vaccine Fund forms part of the landscape of international public entities, it is supported in large part by private philanthropic contributions [1]. By improving federal budgetary allocation and per-capita health care spending there is exponential potential to increase vaccine funding. These components combined with short term low interest rate loans from the World Bank, and improving Federal-State government co-financing (using state population density: income ratios) may help cover the impending funding gap. Our modest simulation analysis demonstrated the potential to raise millions of dollars just from one possible tax source; when combined with the revenue from other recommended sources the amount raised will potentially surpass Nigeria’s long-term financing needs. Since Oil and Gas services account for about a tenth of our GDP, there are numerous potential options for revenue in other service areas. It is apparent that the future funding source is not clear and there is urgent need for long term financing options to provide 100% self-funding of the NIP between 2020 and 2025 (as it is the expectation that foreign aids and dollar contributions will be unavailable by 2020). The ROI of vaccine in terms of averted illnesses, productivity, improved quality of life and Years of Potential Lives Lost supersedes all other considerations and should prompt urgent activities to cover the impending finance coverage gap.

## Competing interests

The authors declare no competing interest.
